# Supplementary material for: Influence of Lenvatinib on the Functional Reprogramming of Peripheral Myeloid Cells in the Context of Non-Medullary Thyroid Carcinoma
Source: Pharmaceutics. 2023 Jan 26;15(2):412. doi: 10.3390/pharmaceutics15020412 (PMC9960916; doi:10.3390/pharmaceutics15020412)
Supplement: Supplementary file 1 [file pharmaceutics-15-00412-s001.zip › pharmaceutics-2131816-Supplementary Material.pdf]

pharmaceutics

Article

Supplementary Material: Influence of Lenvatinib on the Functional Reprogramming of Peripheral Myeloid Cells in the Context of Non-Medullary Thyroid Carcinoma

Chunying Peng , Katrin Rabold , Mihai G. Netea , Martin Jaeger and Romana T. Netea-Maier

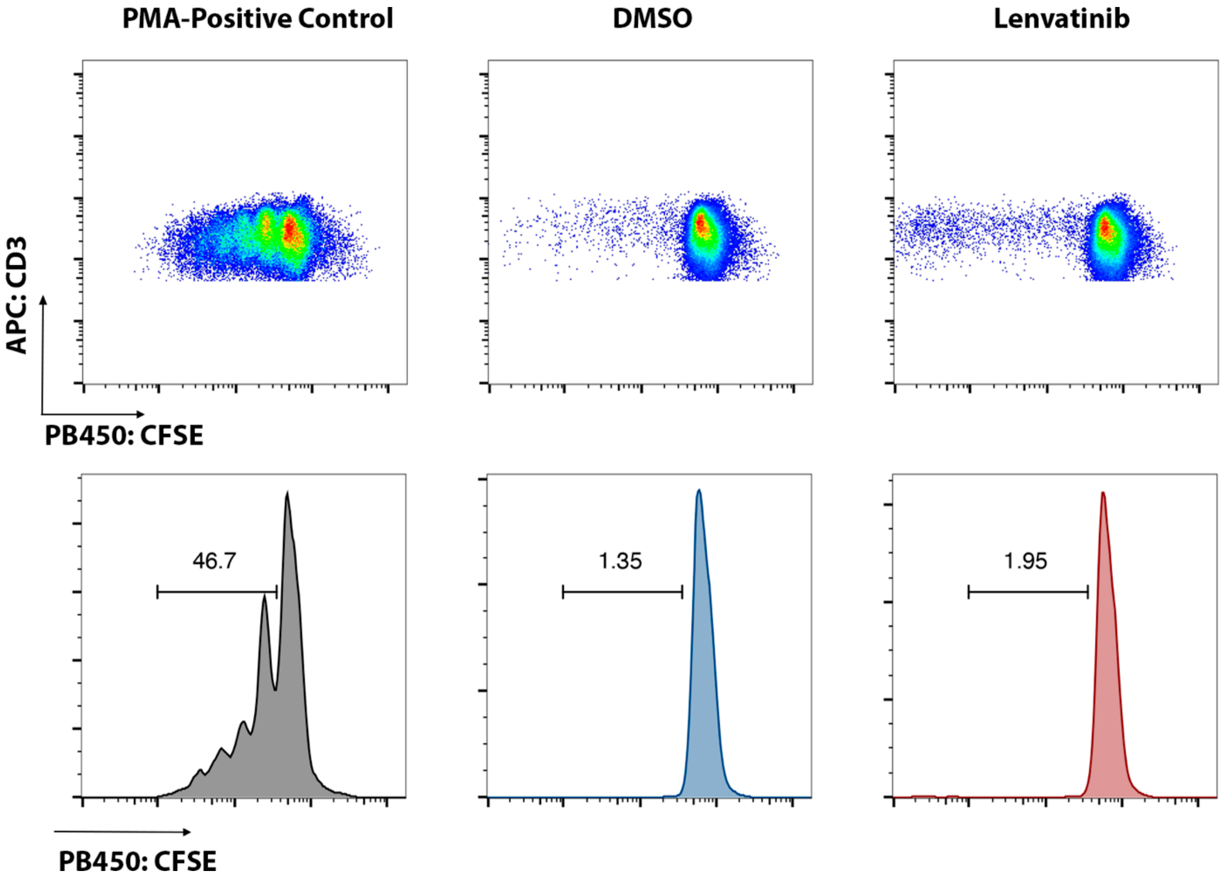

Figure S1. T cell proliferation in the presence of Lenvatinib. CFSE-labeled PBMCs were incubated with Lenvatinib (1µM) or DMSO control for 7 days. PMA-activated PBMCs were used as a positive control. Viable CD3⁺ cells were gated for analysis. The upper and lower panel shows the dot plot and histogram from one representative donor. Numbers on histograms represent the percentage of proliferating T cells, n = 3. CFSE, carboxyfluorescein succinimidyl ester.

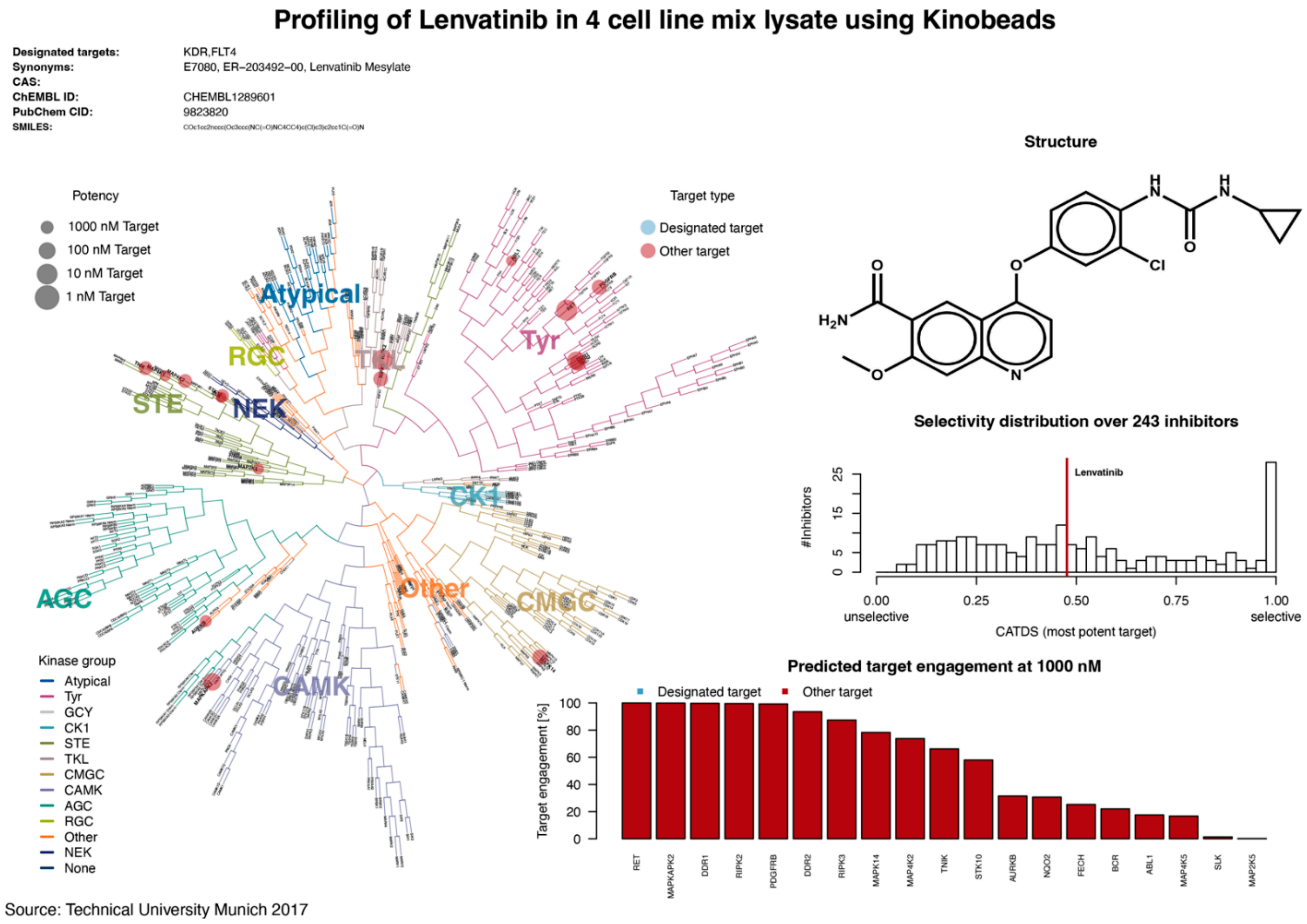

Figure S2. Target landscape of Lenvatinib in 4 cell lines. The figure was downloaded from the public database ProteomeXchange (http://www.proteomexchange.org). The upper-right figure shows the chemical structure of Lenvatinib. A phylogenetic tree of all kinases for the four cell lines (K-562, MV-4-11, SK-N-BE, COLO 205) is shown on the left side. The circle size indicates potency; the color code specifies kinase–drug interaction with Lenvatinib designated or other targets. The drug selectivity is shown as CATDS (concentration and target-dependent selectivity) value. The lower right figure shows the predicted target engagement of Lenvatinib at 1000nM.

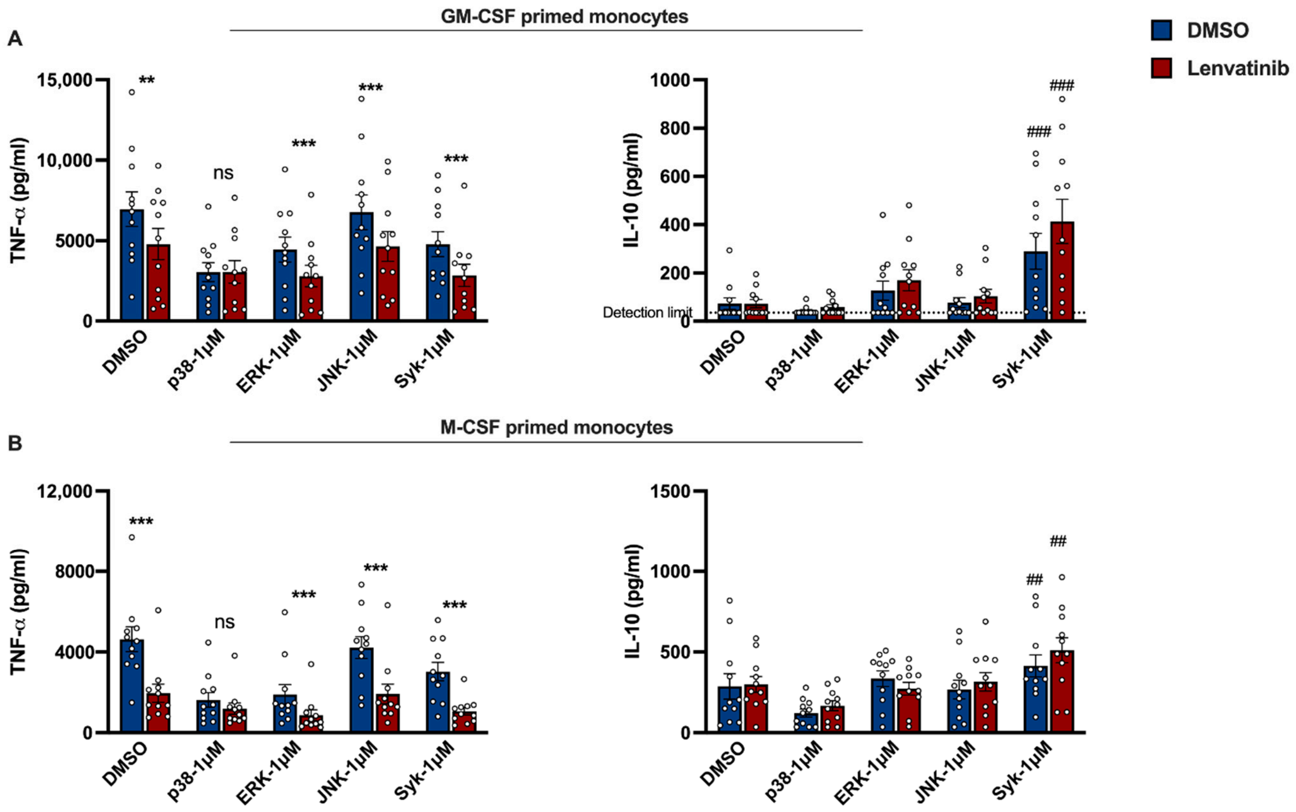

Figure S3. The impact of TLR4 downstream kinase inhibitors on Lenvatinib-mediated immunomodulation in GM-CSF/M-CSF-primed monocytes. Monocytes were primed with GM-CSF (10ng/ml) or M-CSF (50ng/ml) in the presence of kinase inhibitor with or without concomitant Lenvatinib treatment. After 24h of incubation, monocytes were stimulated with LPS and cytokine production was determined by ELISA. (**A**) Cytokine response of GM-CSF primed monocytes, n = 12. (**B**) Cytokine response of M-CSF primed monocytes, n = 12. The bar graphs indicate mean ± SME; each dot represents one healthy donor. ** $p < 0.01$, *** $p < 0.001$ when single kinase inhibitor was compared with kinase inhibitor plus Lenvatinib. ## $p < 0.01$, ### $p < 0.001$ when kinase inhibitor was compared with DMSO by nonparametric Wilcoxon matched-pair test.
